# Optimized Solutions of Electrocardiogram Lead and Segment Selection for Cardiovascular Disease Diagnostics

**DOI:** 10.3390/bioengineering10050607

**Published:** 2023-05-18

**Authors:** Jiguang Shi, Zhoutong Li, Wenhan Liu, Huaicheng Zhang, Qianxi Guo, Sheng Chang, Hao Wang, Jin He, Qijun Huang

**Affiliations:** 1 School of Physics and Technology, Wuhan University, Wuhan 430072, China; shijig@whu.edu.cn (J.S.); whliu@whu.edu.cn (W.L.); zhuaicheng@whu.edu.cn (H.Z.); inge_2017@whu.edu.cn (Q.G.); changsheng@whu.edu.cn (S.C.); wanghao@whu.edu.cn (H.W.); jin.he@whu.edu.cn (J.H.); 2 Huangpu Branch of Shanghai Ninth People’s Hospital, Shanghai Jiaotong University School of Medicine, Shanghai 200011, China; captain777@126.com

**Keywords:** electrocardiogram (ECG), Genetic Algorithm-Based ECG Leads and Segment Length Optimization (GA-LSLO) framework, portable ECG detection devices, cardiovascular disease detection

## Abstract

Most of the existing multi-lead electrocardiogram (ECG) detection methods are based on all 12 leads, which undoubtedly results in a large amount of calculation and is not suitable for the application in portable ECG detection systems. Moreover, the influence of different lead and heartbeat segment lengths on the detection is not clear. In this paper, a novel Genetic Algorithm-based ECG Leads and Segment Length Optimization (GA-LSLO) framework is proposed, aiming to automatically select the appropriate leads and input ECG length to achieve optimized cardiovascular disease detection. GA-LSLO extracts the features of each lead under different heartbeat segment lengths through the convolutional neural network and uses the genetic algorithm to automatically select the optimal combination of ECG leads and segment length. In addition, the lead attention module (LAM) is proposed to weight the features of the selected leads, which improves the accuracy of cardiac disease detection. The algorithm is validated on the ECG data from the Huangpu Branch of Shanghai Ninth People’s Hospital (defined as the SH database) and the open-source Physikalisch-Technische Bundesanstalt diagnostic ECG database (PTB database). The accuracy for detection of arrhythmia and myocardial infarction under the inter-patient paradigm is 99.65% (95% confidence interval: 99.20–99.76%) and 97.62% (95% confidence interval: 96.80–98.16%), respectively. In addition, ECG detection devices are designed using Raspberry Pi, which verifies the convenience of hardware implementation of the algorithm. In conclusion, the proposed method achieves good cardiovascular disease detection performance. It selects the ECG leads and heartbeat segment length with the lowest algorithm complexity while ensuring classification accuracy, which is suitable for portable ECG detection devices.

## 1. Introduction

According to the latest research from the World Health Organization, cardiovascular diseases (CVDs) are the leading cause of death worldwide [1]. Statistics indicate that approximately 17.9 million people died of cardiovascular disease in 2019, accounting for 32% of global deaths. Therefore, the diagnosis of CVDs has an important role. Electrocardiogram (ECG) reflects the electrical activity of the heartbeat cycle, which is an important tool for the diagnosis of cardiovascular disease. The most commonly used ECG contains 12 leads: limb leads (I, II, III, avR, avL, avF) and chest leads (V1, V2, V3, V4, V5, V6). Cardiologists analyze the patient’s condition based on these multi-lead ECGs. However, it takes a lot of time and effort for cardiologists to manually diagnose with an ECG, so an accurate and efficient automatic multi-lead ECG diagnosis technology is urgently needed.

With the development of artificial intelligence, automatic analysis of multi-lead ECG based on machine learning has attracted the interest of researchers. Traditional machine learning methods mainly include two stages: feature extraction and classification. Shi et al. [2], Alim and Islam [3], and Shen et al. [4] extracted various manual features such as RR interval, morphology features, average QRS interval, average QTC interval, and ST-segment to detect cardiovascular diseases. In addition, Khorrami et al. [5], Desai et al. [6], and Raj et al. [7] used discrete cosine transform (DCT) and discrete wavelet transform (DWT) for feature processing, while Zhao et al. [8], Martis et al. [9], and Kanaan et al. [10] used principal component analysis (PCA) for feature dimensionality reduction, which can further improve the quality of extracted features. As for the classification stage, it is crucial to choose the appropriate classifier. Uyar et al. [11] and Chauhan et al. [12] used logistic regression (LR), Shen et al. [4], Kanaan et al. [10], Padhy et al. [13], and Han et al. [14] used support vector machine (SVM), Sahoo et al. [15] and Park et al. [16] used decision tree, and Yang et al. [17], Dilmac et al. [18], and Sun et al. [19] used k-nearest neighbor (KNN) as an automatic classifier and achieved acceptable results in the classification of cardiac diseases such as myocardial infarction [13,14,19] and arrhythmia [5,11,12,18]. The above-mentioned feature extraction-based methods may achieve automatic detection of cardiovascular disease, but they also have several shortcomings. The results of the above methods are dependent on the quality of the extracted features. The classification process requires manual intervention and relies heavily on the medical knowledge of the experimenter.

The above drawbacks can be overcome in deep learning (DL). DL can learn useful features from raw data without requiring extensive data preprocessing, feature engineering, or handcrafted rules, making it particularly suitable for interpreting ECG data [20]. Currently, the convolutional neural network (CNN) is the most commonly used deep learning algorithm. With the advancement of CNNs, automatic detection algorithms for cardiac disease based on a single lead, multiple leads (fewer than 12 leads), and all 12 leads have been widely developed. Kharshid et al. [21] implemented atrial fibrillation detection by using single-lead ECG. Acharya et al. [22] and Xiaolin et al. [23] detected myocardial infarction and five arrhythmias based on lead II, respectively. However, single-lead ECG carries limited information, resulting in insufficient detection accuracy. Reasat et al. [24] used three-lead (II, III, and avF) ECG signals to diagnose myocardial infarction. Liu et al. [25] used leads V2, V3, V5, and avL to detect generalized anterior myocardial infarction. Zhang et al. [26] proposed a CNN-based multi-lead branch fusion network (MLBF-Net) architecture, which achieves an average F1 score of 0.855 in the classification of nine types for arrhythmia by using twelve-lead signals. Ye et al. [27], Yang et al. [28], and Baloglu et al. [29] also obtained acceptable results in cardiac detection based on 12-lead ECG. Jekova et al. [30] explored the effect of different ECG lead combinations on disease detection. Each single lead and different lead combinations were used to detect atrial fibrillation and achieved good results. However, for multiple leads, the classification algorithm may not be universal. For example, in [24], leads II, III, and avF performed well in the detection of MI, but may not achieve satisfactory results when applied to arrhythmias. For all 12 lead methods, complex algorithms are inevitably required, which do not meet the requirements of portable devices.

Another issue worth noting is ECG segmentation. In reality, the time length of the original ECGs collected by the ECG machine is not fixed, so a reasonable signal segmentation method is critical for disease detection. Reasat et al. [24] segmented the original signal into short segments (196 samples) of 3.072 s. Ye et al. [27] extracted ten 6 s segments from each ECG recording and then stacked them. Hussein et al. [31] performed experiments using 1 min long ECG segments. Krasteva et al. [32] analyzed the effect of a 2 s to 10 s duration on performance in the detection of shockable (Sh) and non-shockable (NSh) rhythms, and the best performance was achieved at 5 s. Obviously, no standard rules for heartbeat segmentation exist. Short ECG segments may miss information, affecting diagnostic results. Too long segments often result in more complex algorithms and greater amounts of computation, which is detrimental to the real-time capability of the algorithm.

With the advancement of medical technology, portable ECG monitoring devices [33,34,35] are constantly developed. For example, Yang et al. [33] have designed a portable ECG acquisition system, which transmits the collected ECG to a cloud platform via Wi-Fi and displays the ECG through a smart terminal. Sun et al. [34] developed a health shirt integrated with ECG electrodes to provide ECG monitoring during exercise, which can diagnose six types of cardiovascular diseases. Liu et al. [35] have designed an IoT-based portable 12-lead ECG monitoring system that can transmit the collected ECGs to a cloud server. For portable CVD diagnostic devices, it is crucial to select appropriate leads and length of ECG signal segments intelligently. Fortunately, the genetic algorithm (GA) has impressive performance in finding optimal solutions [36] and is inspired by biological evolutionary processes to optimize populations through selection, crossover, and mutation to generate high-quality optimal solutions.

The objective of this study was to automatically generate optimal ECG lengths and lead combinations for different disease classification tasks while balancing classification performance and algorithm complexity. Specifically, the validation was performed on the SH database and PTB database [37] to achieve efficient arrhythmia and myocardial infarction detection, respectively. Moreover, a Raspberry Pi was used to explore the effectiveness of the proposed method in terms of hardware implementation. 

## 2. Materials and Methods

### 2.1. Datasets

The experiments in this article verify the algorithms by arrhythmia and myocardial infarction detection. The arrhythmia data are from the non-public SH database and the myocardial infarction data are from the public PTB database. The details of the two databases are as follows: 

#### 2.1.1. The SH Database

The data come from the Cardiology Department of Huangpu Branch of Shanghai Ninth People’s Hospital, preserving most of the original ECG signals collected by the hospital, which makes the experiment more generalizable. The SH database provides 75, 111 12-lead ECG records. The length of the records varies from 11 s to 92 s, and the sampling rate is 1000 Hz. Each record is overall diagnosed and labeled by a professional cardiologist, and the diagnosis includes 46 types, such as normal ECG, atrial premature beats, tachycardia, etc. The identification information of each patient is removed to preserve personal privacy, only the ECG and diagnostic results are retained. For this experiment, five of the most common signals with a relatively high number are selected, including normal ECG (N), premature atrial contractions (PAC), premature ventricular contractions (PVC), sinus tachycardia (T, sinus heart rate more than 100 beats per minute), and sinus bradycardia (B, sinus heart rate less than 60 beats per minute). The datasets in the experiments are divided according to the inter-patient paradigm, i.e., data from the same patient will not be present in both training and test sets. We randomly select 80% of the patients to constitute the training set and the remaining 20% are used as the test set. Table 1 shows the quantitative information for each type of ECG signal and the number of patients in the training and test sets. 

#### 2.1.2. The PTB Database

The PTB database [38] is an open-source database provided by the National Metrology Institute of Germany. It contains 549 records from 290 subjects. The length of the records is not fixed, ranging from 32 s to 120 s, and the sampling rate is 1000 Hz. The records are collected and diagnosed by cardiologists. The PTB database includes nine diagnostic categories, such as myocardial infarction, cardiomyopathy, dysrhythmia, healthy controls, etc. In this study, standard 12-lead ECGs from 148 myocardial infarction subjects and 52 normal subjects in the PTB database are used for research. The dataset is divided into training and test sets using the same paradigm employed for the SH database. Detailed information is shown in Table 2. 

### 2.2. The Genetic Algorithm-Based ECG Leads and Segment Length Optimization Framework

**Figure 1:**
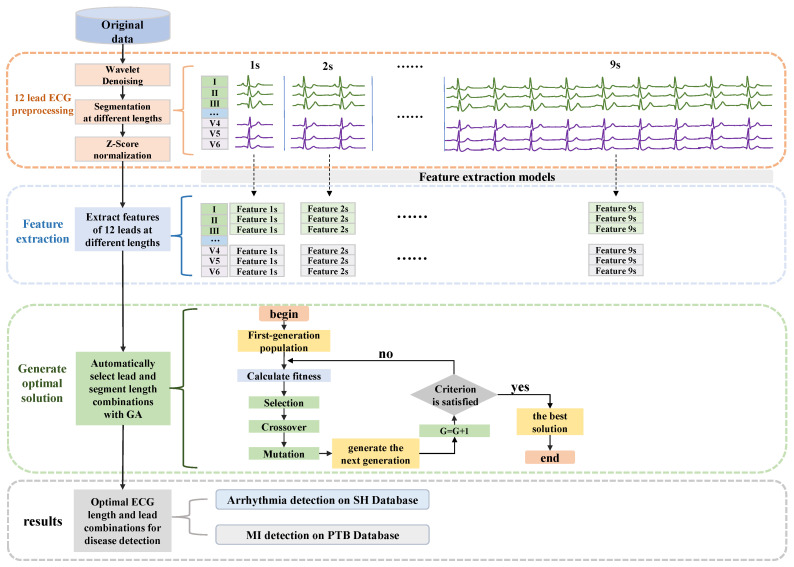
The overall structure of the proposed method. Contains four parts: ECG preprocessing, feature extraction of 12-lead ECG signals in different segments, GA-based algorithm to generate optimal solutions, and final classification results.

As shown in Figure 1, the method proposed in this paper can be mainly divided into four parts: the first one is the original signal preprocessing, which includes signal denoising, signal segmentation to different lengths, and signal normalization. The second is to extract the features of 12 leads separately under different segment lengths. Then, the optimal solutions for the combination of ECG leads and segment length are automatically generated by a GA-based algorithm. Finally, the final classification results can be obtained. Detailed descriptions of each part are as follows.

#### 2.2.1. Raw ECG Data Preprocessing

Electrocardiograms record the electrical activity of the heart. Due to the bad contact between the electrode and the body, the subject’s muscle activity, etc., the collected ECG signals inevitably contain noise, such as baseline wandering, electromyogram noise, etc. [39]. These noises affect the detection results of heart disease. The advantages of wavelet transform in ECG signal denoising have been demonstrated, and the Daubechies 6 wavelet transform [40] is used for denoising in this study.

Since the length of the original ECG data is not fixed, but the input of our network models requires fixed-length heartbeat segments, the original signal needs to be segmented. In this study, to explore the effect of different fragment lengths on disease detection, as shown in Figure 1, nine segmentation types are used to cut the original signal into fragments of 1 s to 9 s, which are respectively input into nine structurally identical networks. The fragments are segmented sequentially from the beginning of the ECG and no additional QRS wave detection is performed, which simplifies the whole algorithm system, reduces the reliance on R-peak detection, and improves the robustness and generality of the system. The statistics of the fragments are shown in Table 3. In the segmentation phase, each fragment is labeled with the same label as the original ECG records. Although some short (such as 1 s, 2 s) fragments may not contain a completely abnormal heartbeat (such as premature atrial contractions (PAC), premature ventricular contractions (PVC)), since the disease exists in the long record, the short fragment may have implied information about an impending abnormality, which can also be captured by the deep learning model as valid information. In addition, to mitigate the effects of baseline offset, all segments are processed using Z-score normalization.

#### 2.2.2. Feature Extraction at Different Fragment Lengths

This section introduces the process of ECG signal feature extraction based on ResNet [41]. Traditional multi-lead classification methods [26,27,29] train all leads simultaneously. In this study, two feature extraction models are designed for arrhythmia and myocardial infarction to extract the features of 12 leads separately with higher quality. 

**Figure 2:**
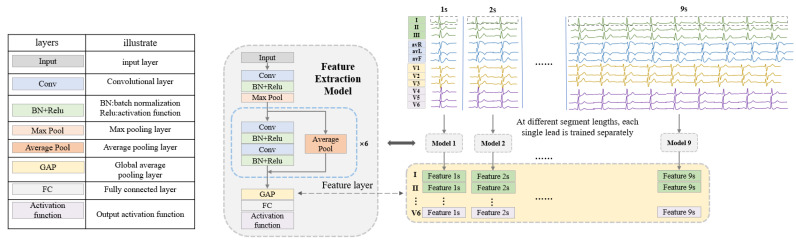
The framework of feature extraction at different fragment lengths.

As shown in Figure 2, the feature extraction model (FEM) is developed on the basis of ResNet. Since each disease and each lead requires feature extraction for nine fragment lengths (1 s to 9 s), for inputs of different sizes, nine networks suitable for segment lengths from 1 s to 9 s are designed by modifying the input layer. Each network contains 13 convolutional layers and the structure and configuration are shown in Table 4. The feature extraction process is performed by the FEM trained on the classification task. For the different classification tasks (arrhythmia and myocardial infarction), the feature extraction models can be obtained by modifying the number of nodes in the fully connected layers and the activation function in the network, respectively. For the SH database, the FEMs are trained on the classification task of normal ECG, PAC, PVC, tachycardia, and bradycardia, so the number of nodes in the fully connected layer is set to 5 and the activation function is Softmax. For the PTB database, the models are trained using a binary classification task of MI and normal signal, so the number of nodes in the fully connected layer is set to 2 and the activation function is sigmoid. Each lead is trained separately at each length to obtain features. Finally, the training set and test set data are re-input into the trained FEM, and the output of the global average pooling (GAP) layer in the network structure is used as the final extracted features. In this way, features for two disease categories (arrhythmia and myocardial infarction) are obtained, each containing 12 leads at 9 ECG lengths. These features can be used directly in later classification, for example, when testing the case of ECG lengths for 3 s and lead combinations for II, avR, V3, and V4, the features of the 4 leads with a heartbeat segment of 3 s are directly selected and concatenated, and then the classifier is used to classify and test the performance.

The cross-entropy loss function is used during network training on the SH database. In addition, for PTB data, since the amount of myocardial infarction data is much larger than that of the healthy control data, the weighted cross-entropy loss function [42] is used to deal with the class imbalance problem. The Adam optimizer is used to reduce the loss [21], and the learning rate is set to 0.001. The batch size is set to 128, and each lead with different segment lengths is trained for 40 epochs.

#### 2.2.3. Generating Optimal Combination by Genetic Algorithm

##### The Proposed Encoding Strategy

**Figure 3:**
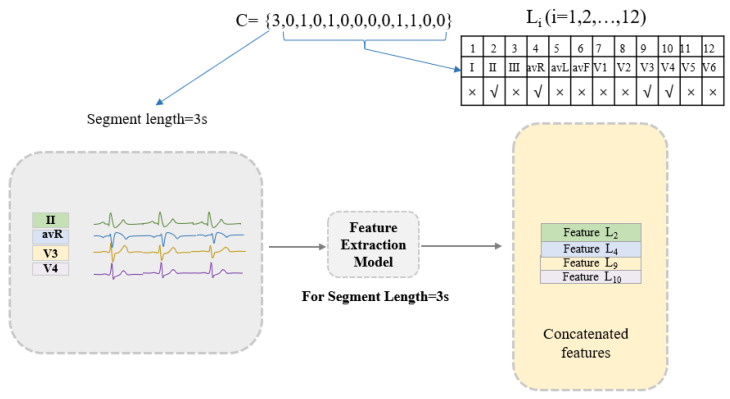
An example of the proposed encoding strategy. Feature extraction model: for the segment length of 3 s, the specific information of the trained ResNet-based neural network structure is described in Section 2.2.2.

In the proposed method, the set C = [*T_L_*, *L_1_*, *L_2_*, *… L_i_ … L_12_*] represents the combination of the time length (*T_L_*) of the heartbeat fragment and the ECG leads (*L_i_*). Among them, *T_L_* = 1,2, …, 9 indicates that the length of the heartbeat is from 1 s to 9 s. *L_i_* = 0 or 1 and, when it is 0, it means that the ECG signal of the *i*th lead is not used, and when it is 1, the ECG signal of the *i*th lead is used. It is worth noting that *L_1_*–*L_12_* cannot be 0 at the same time, which means 0 leads are selected for classification. The combination of ECG leads and heartbeat segment length can be determined by C. Disease classification is performed using the features of the selected leads (leads with *L_i_* = 1) at the selected lengths (length = *T_L_*) extracted by the feature extraction model. As shown in Figure 3, taking C = [3,0,1,0,1,0,0,0,0,1,1,0,0] as an example, the length of the heartbeat fragment is 3 s, and the features of leads II, avR, V3, V4 are selected and concatenated for further classification.

Theoretically, the best solution can be given by running all the possible values of set C, but this non-GA-based approach inevitably takes a lot of time. The genetic algorithm has a great advantage in seeking solutions, so this study uses the GA to reduce the time of searching for the optimal solution. The following experiments verify the effectiveness of the proposed algorithm.

##### Classification Algorithm Combined with the Lead Attention Module

**Figure 4:**
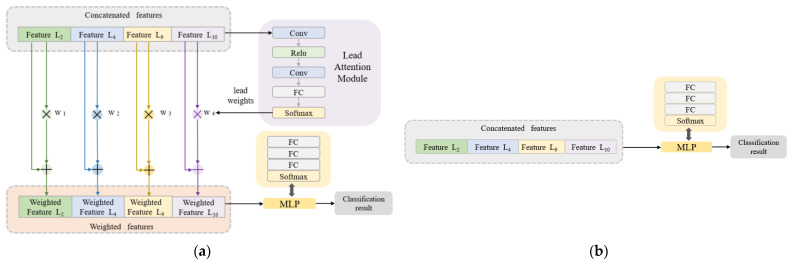
Classification network structure. (**a**) The algorithm combined with LAM. (**b**) The pure MLP-based algorithm.

In the classification stage, the concatenated features are used as the input data of the classification network. An innovative lead attention module (LAM) is proposed. The LAM is inspired by the channel attention module [43] and it is updated based on the ECG lead properties. As shown in Figure 4a (still taking C = [3,0,1,0,1,0,0,0,0,1,1,0,0] as an example), the LAM is composed of a convolutional layer, fully connected layer, and activation function. The number of FC layer nodes in the LAM is set to be the same as the number of leads (the number of leads in the example is 4, then the number of FC layer nodes is 4), so the Softmax layer will output the same number of weights as the selected number of leads. The features from each lead are multiplied by their respective weights and added to the original features to obtain the lead-weighted features. Then, a multi-layer perceptron (MLP) composed of fully connected layers and activation functions is used for the final disease classification. Compared with the pure MLP-based classification algorithm in Figure 4b, the classification algorithm combined with the LAM can effectively capture the dependencies between ECG leads and improve the classification effect of the network.

##### Generating the Optimal Solutions

This section describes the process of generating the optimal solutions for the combination of segment lengths and ECG leads.

The genetic algorithm (GA) is a global optimization method that originated from computer simulations performed on biological systems. It simulates the natural selection, crossover, and mutation that occur in genetics. The genetic algorithm starts from a random initial population and produces individuals more adapted to the environment through selection, crossover, and mutation operations. The population evolves toward a better search space. Moreover, it iterates continuously and finally converges on the most adapted individual to find the optimal solution to the problem. In this paper, each individual is represented by a C defined above, corresponding to a combination of the heartbeat segment and the ECG leads. Algorithm 1 gives the algorithm framework, mainly including initialization, fitness calculation, selection, crossover and mutation, and iterative processes. The detailed introduction is as follows.
**Algorithm 1** Generation of optimal ECG lengths and lead combinations based on GA**Input**: Feature data of each lead with different segment lengths extracted in Section 2.2.2. Algorithm settings, population size = 100, maximum number of iterations = 20 **Output**: Optimal combination of ECG leads and segment length1 G_0_: number of iterations: *i* = *0*. Initialize the population with the given population size using the proposed encoding strategy. 2 **for** *i = 0, 1, 2, …, 20* **do** 3  Calculate the fitness of each individual in the population G_i_ 4  Select the individuals with the top 50 fitness as the parent5  Generate Gi by the selected parents using crossover and mutation operations6  *i = i + 1*7  **if** the maximum fitness in the population remains unchanged for three generations8    break from step 29  **else** 10     continue the iteration11 **end**12 **Return** the individual with the maximum fitness in the iterative process

In the initialization phase of this experiment, a uniformly distributed population is randomly generated, and the size of the population is set to 100.

In the fitness calculation phase, the combination of heartbeat segments and ECG leads represented by each individual is evaluated using the classification algorithm introduced above to obtain the classification accuracy (*Acc*) and F1 score (*F1*) (large *F1* and *Acc* represent good classification performance). Considering the impact of the lead number used in the classification and the length of the heartbeat segment on the algorithm complexity, the fitness formula is given as:

(1) $$\mathrm{fitness}=\alpha \times \;F1+\beta \times \;\mathrm{Acc}-\;\gamma \times \;T_{L}-\;\sigma \times \;\sum_{i=1}^{i=12}Li$$

where *F1* and *Acc* represent the F1 score and classification accuracy, respectively. *T_L_* and *Li* are defined in the proposed encoding strategy. The principle of the parameter settings here is to first ensure the results of disease detection, and on this basis, preferentially select individuals with low algorithm complexity for application on the portable ECG detection systems. After several experimental adjustments, *α*, *β*, *γ*, and *σ* are set to 1, 1, 0.002, and 0.01, respectively.

In the selection stage, each individual in the population uses Formula (1) to calculate their fitness, and then the top 50 individuals in terms of fitness are selected as the parents of the next generation, which can retain the individuals with high quality in the population.

**Figure 5:**
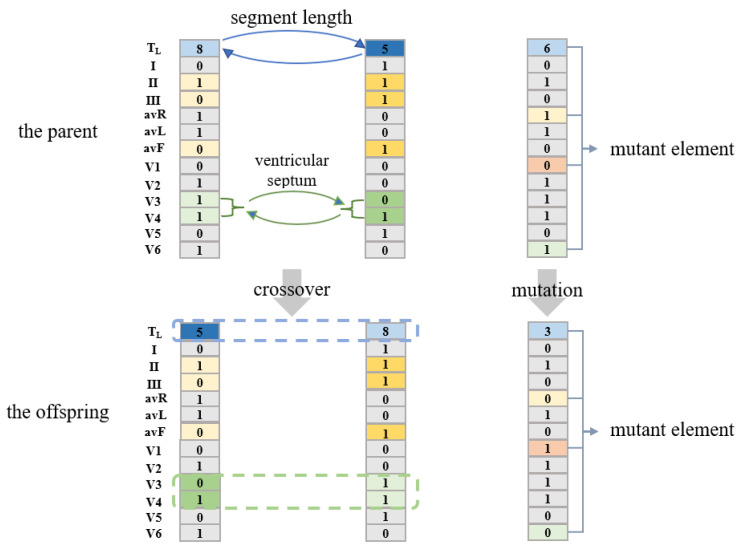
Examples of crossover and mutation.

Then, the next population is generated through crossover and mutation operations. ECG leads can reflect the heart parts [44]. For the crossover operation, two individuals in the parent generation are randomly selected, and the crossover is carried out according to the heart parts reflected by the leads in Table 5. {[*T_L_*], [*L_1_, L_5_*], [*L_2_, L_3_, L_6_*], [*L_4_*], [*L_7_, L_8_*], [*L_9_, L_10_*], [*L_11_, L_12_*]} are crossed according to the probability of [0.8, 0.5, 0.5, 0.5, 0.5, 0.5, 0.5]. By exchanging the lead information of the 6 heart parts of the two parent individuals, the parents’ information from the 6 critical groups (shown in Table 5) can be retained. Twenty groups of crossover operations can generate forty offspring individuals. For the mutation operation, 10 individuals in the parent generation are randomly taken, and 13 elements [*T_L_*, *L_1_*, *L_2_*, *… L_11_*, *L_12_*] in each individual are mutated with probability [0.8, 0.5, 0.5, … 0.5, 0.5]. That is, for the first element *T_L_*, it changes to other fragment lengths with a probability of 0.8, and for the 2nd element to the 13th element, they mutate to the opposite value with a probability of 0.5 (0 to 1, 1 to 0). Figure 5 shows examples of crossover and mutation.

Population iteration is achieved through the above selection, crossover, and mutation operations. The population evolves in the direction of increasing overall fitness. The maximum number of iterations set in the experiment is 20, and in order to improve the efficiency of the algorithm, when the maximum fitness in the population does not change for three consecutive generations, the iteration is terminated. The individual with the greatest fitness in the entire iterative process can be obtained, which is the optimal solution.

#### 2.2.4. Performance Metrics

This study comprehensively evaluates the final disease detection effect by calculating sensitivity (*Sen*), specificity (*Spe*), positive predictivity (*Ppr*), accuracy (*Acc*), and F1 score (*F1*). The calculation formula is as follows:

(2) $$Sen=\frac{TP}{TP+FN}$$

(3) $$Spe=\frac{TN}{TN+FP}$$

(4) $$\;Ppr=\frac{TP}{TP+FP}$$

(5) $$Acc=\frac{TP+TN}{TP+TN+FN+FP}$$

(6) $$F1=\frac{2\times Sen\times Ppr}{Sen+Ppr}$$

where *TP*, *TN*, *FP*, and *FN* represent true positive, true negative, false positive, and false negative, respectively. In the evaluation of disease detection performance, these metrics are calculated separately for each category.

### 2.3. The Hardware Implementation of the Algorithm

**Figure 6:**
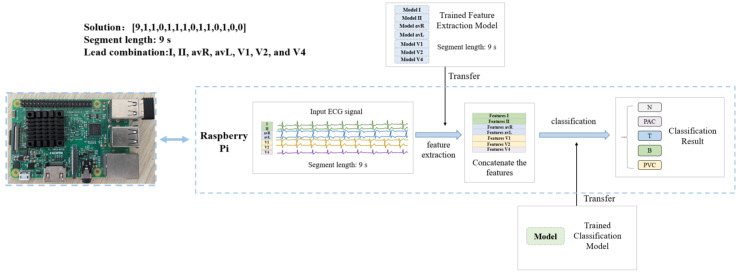
Structure of arrhythmia detection device based on Raspberry Pi.

To verify the convenience of the proposed algorithm in hardware implementation, cardiac disease detection devices are fabricated using Raspberry Pi 3 Model B. The Raspberry Pi is small in size and easy to carry and can be used to simulate a portable ECG device. As shown in Figure 6 (taking the solution [9,1,1,0,1,1,1,0,1,1,0,1,0,0] for arrhythmia detection as an example), for the arrhythmia and myocardial infarction detection devices, the feature extraction model trained in Section 2.2.2 and the classification model trained in Section 2.2.3 are transferred to the Raspberry Pi. The input ECG is feature extracted and classified using the optimal solution of ECG length and lead combinations generated in Section 2.2.3. 

## 3. Results

### 3.1. Arrhythmia Detection in SH Database

Through the method combined with the GA proposed in this paper, the final generated solution for arrhythmia detection in the SH database is C_1_ = [9,1,1,0,1,1,0,1,1,0,1,0,0]. This means for the detection of arrhythmia in the SH database, after evaluation of algorithm complexity and classification efficiency through the GA, the optimal segment length is 9 s and the optimal lead combination is seven leads (I, II, avR, avL, V1, V2, and V4). The final classification results are shown in Table 6 and Table 7.

The proposed method achieves an accuracy of 99.65% (95% confidence interval: 99.20–99.76%) for arrhythmia detection in the SH database using seven ECG leads. Clinically, P-waves are key to the diagnosis of PAC and PVC. Leads II and V1 reflect the P-wave most clearly, so these two leads are the most commonly used for arrhythmia analysis. The optimal solution given by the proposed method includes leads II and V1, which is consistent with the clinical diagnostic criteria. More importantly, leads I, avR, avL, V2, and V4 are also included in the optimal solution. It can be speculated that these five leads (I, avR, avL, V2, and V4) are also critical for the diagnosis of arrhythmia, which has guiding significance for doctors to analyze arrhythmia.

### 3.2. MI Detection in PTB Database

For MI detection in the PTB database, the final generated solution is C_2_ = [5,1,0,1,0, 0,1,0,1,0,1,0,1]. It shows for the detection of MI in PTB, after evaluating the algorithm complexity and classification efficiency through the GA, the optimal segment length is 5 s, and the optimal lead combination is six leads (I, III, avF, V2, V4, and V6). The final detection results are shown in Table 8 and Table 9.

It can be seen that the proposed method achieves an accuracy of 97.62% (95% confidence interval: 96.80–98.16%) in MI detection. Although the sensitivity and F1 of healthy controls are lower than the myocardial infarction, the result is acceptable due to the lower number of HC than MI and the weighted cross-entropy has been used in the experiment to mitigate the category imbalance. According to [45], six leads (I, III, avF, V2, V4, and V6) are critical for the detection of MI, which proves the theoretical medical significance of the proposed method. In general, the proposed method uses six leads to achieve effective detection of myocardial infarction. It ensures the detection results while selecting the algorithm with the lowest complexity, indicating the efficiency of automatic lead and segment length optimization. The inter-patient paradigm makes the results more clinically meaningful.

### 3.3. The Comparison of Lead Selection Methods

The algorithm automatically generates combinations of leads and segment lengths through a GA-based approach. The optimal solutions given in the SH database and PTB database are C_1_ = [9,1,1,0,1,1,0,1,1,0,1,0,0] and C_2_ = [5,1,0,1,0,0,1,0,1,0,1,0,1], respectively. In order to verify the validity of the solution, 12 single-lead ECG data and all 12 lead ECG data are used for comparative experiments under the same segment length as C_1_ and C_2_. The datasets for the experiments are partitioned in the same way as in Section 3.1 and Section 3.2. The features of each lead can be obtained from the feature extraction stage, the features are input into the classification model (LAM+MLP), and they are retrained to be suitable for single-lead and all 12 lead classification (for all 12 leads, the features are concatenated first). The experimental results are shown in Table 10 and Table 11. Due to the large number of results, the results of multi-leads (fewer than 12 leads) are not listed in the table, but multi-lead cases (fewer than 12 leads) have been screened during the iteration of the genetic algorithm.

According to Table 10 and Table 11, the GA-based approach is attractive in the optimization of ECG lead selection. The proposed method achieves the best classification results on both the SH database and the PTB database. Especially in the PTB database, the advantage of MI detection is relatively obvious. Compared with the single-lead-based methods, the proposed method has more accurate detection results, and both the accuracy and F1 score are greatly improved. Compared with the method based on all 12 ECG leads, the proposed method achieves an optimized scheme with suitable length and ECG leads. This verifies that it is not true that the more ECG leads, the better the classification effect. The detection method based on all 12 leads has information redundancy, and the redundant information of ECG leads will affect the classification results. Furthermore, fewer leads correspond to less complex algorithms, proving the practical significance of this work. In conclusion, the method proposed in this paper can reduce the algorithm complexity while ensuring the results of disease detection.

### 3.4. The Performance of the Algorithm with a Fixed Lead Number

In portable medical devices, some users may require a fixed number of leads. This section takes the limited numbers of leads of two, three, and four as examples to analyze the performance of the algorithm. For the SH database and the PTB database, under the same segment length of C_1_ and C_2_ (the optimal solution given in Section 3.1 and Section 3.2), the optimal lead combinations with a fixed total number of leads of two, three, and four are generated through GA iteration. The experimental dataset is divided in the same way as in Section 3.1 and Section 3.2. The experimental results are shown in Table 12.

As shown in Table 12, for arrhythmia detection in the SH database, the heartbeat segment length is fixed at 9 s (consistent with C_1_). The best lead combinations based on two, three, and four leads are included in the optimal solution. It is worth noting that lead V1 is not included in these lead combinations, which is slightly different from the clinical theory. This may be because other leads in the optimal solution (such as leads I, avR, and V4) carry critical information, but are difficult to identify manually. For the neural network model, the characteristics of this information carried by these leads are obvious. These leads are also critical for disease analysis and require physician attention when diagnosing arrhythmias. For MI detection in the PTB database, the heartbeat segment length is fixed at 5 s (consistent with C_2_). The best lead combinations based on two, three, and four leads are included in the optimal solution. This experiment shows that the proposed method can accurately find the most critical leads for disease detection, which further proves the effectiveness of the lead optimization algorithm.

For the classification results, the accuracy is slightly lower than the optimal solution since the number of leads used is less than the optimal solution. However, the detection accuracies of above 90% for arrhythmia and above 95% for MI are acceptable. In conclusion, the proposed method can generate optimal lead combinations for different diseases with a fixed total lead number, indicating its strong flexibility. In addition, it has flexible guiding significance for the hardware implementation system. For example, if a wearable device requires three leads, the proposed method can generate the best lead combination based on the three leads. This further addresses the need for portable medical devices.

### 3.5. The Results of Ablation Experiments

#### 3.5.1. The Effect of the Lead Attention Module

**Figure 7:**
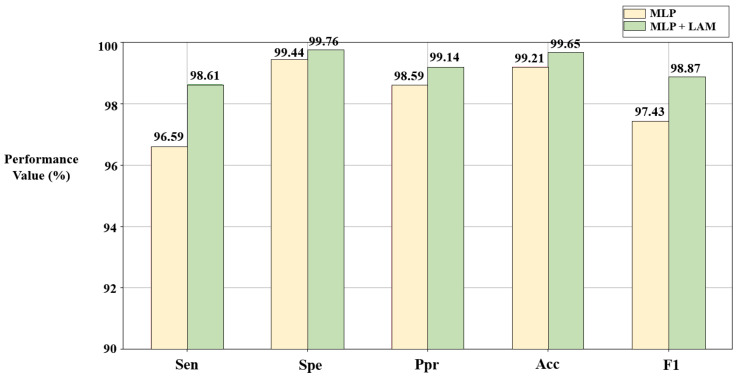
Results of ablation experiments on LAM in the SH database. C_1_ = [9,1,1,0,1,1,0,1,1,0,1,0,0].

**Figure 8:**
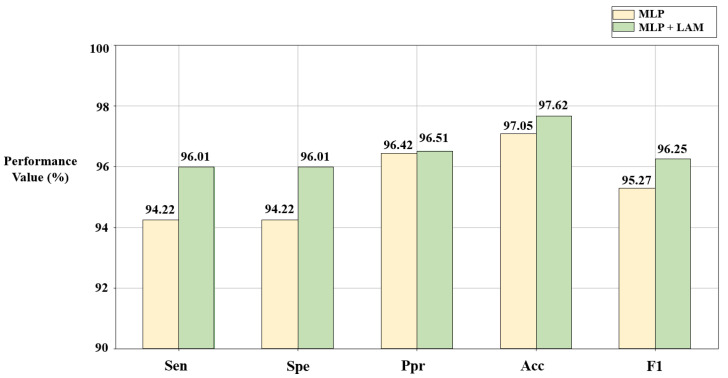
Results of ablation experiments on LAM in the PTB database. C_2_ = [5,1,0,1,0,0,1,0,1,0,1,0,1].

The lead attention module (LAM) can capture the dependencies between ECG leads and improve the classification effect. However, it is still necessary to verify the advantages of the LAM through ablation experiments. In this section, based on the solutions C_1_ and C_2_ given in Section 3.1 and Section 3.2, disease detection is described using the algorithm combined with the LAM (Figure 4a) and the pure MLP-based algorithm (Figure 4b), respectively. The results are shown in Figure 7 and Figure 8.

According to Figure 7 and Figure 8, compared with the pure MLP algorithm, the proposed algorithm combined with the LAM has advantages for arrhythmia detection in the SH database and MI detection in the PTB database, both of which have improvement in *F1*. For the electrocardiogram, not every lead carries the key information for disease detection, and there may be redundant information. The LAM can automatically generate lead weights to weight selected ECG leads, helping the model to highlight key leads, which has a positive effect on disease detection.

#### 3.5.2. The Effect of the Weighted Cross-Entropy Loss Function on PTB Database

In this section, the effect of the weighted cross-entropy loss function on MI detection is analyzed experimentally. Based on the solution C_2_ (ECG length of 5 s, six leads) given in Section 3.2, this section shows the comparison of myocardial infarction detection results using weighted cross-entropy and standard cross-entropy. The results are shown in Table 13 and Table 14. It can be seen from the tables that compared with the standard cross-entropy, the weighted cross-entropy reduces the probability of the prediction model incorrectly predicting HC as the MI class, which improves the overall performance of the MI detection model.

### 3.6. The Results of Model Cross-Checking

In this section, the proposed models are cross-checked, i.e., the arrhythmia model is used to test HC and MI from the PTB database, and the MI model is used to test N, PAC, T, B, and PVC from the SH database. The results of the cross-check are shown in Table 15 and Table 16.

According to the tables above, the accuracy of HC detection using the arrhythmia model is high, i.e., most of the HC data are predicted to be N, which is because the N and HC data are normal signals with high similarities in waveforms. When testing MI data using the arrhythmia model, a large number of MI data are classified as tachycardia (T) and premature ventricular contractions (PVC), which is also in accordance with medical principles because myocardial infarction often leads to increased adrenaline tone which causes tachycardia. In addition, patients with myocardial infarction tend to develop PVC as well [45]. When testing the data from the SH database using the MI model, the vast majority of N are classified as HC, which is reasonable. Moreover, the large majority of T and PVC are classified as MI, which may be due to the fact that myocardial infarction and some arrhythmias are interconnected. In conclusion, MI and arrhythmias are not mutually exclusive, and MI patients often have different arrhythmias (e.g., tachycardia, premature ventricular beats, etc.), so MI may also contain arrhythmia features, which are easily misclassified by the neural network. However, the results of model cross-checking are consistent with clinical theory, which validates the performance of the proposed method.

### 3.7. The Results of Hardware Implementation of the Algorithm

For the arrhythmia detection device (optimal solution: [9,1,1,0,1,1,0,1,1,0,1,0,0]), the feature extraction models trained in Section 2.2.2 for leads I, II, avR, avL, V1, V2, and V4 with segment length of 9 s are transferred to the Raspberry Pi. The features of the input signals of these seven leads are extracted and concatenated by the Raspberry Pi. The corresponding classification model trained in Section 2.2.3 is also transferred to the Raspberry Pi to classify the concatenated features and give the classification results. For the MI detection device (optimal solution: [5,1,0,1,0,0,1,0,1,0,1,0,1]), the leads used for classification are I, III, avF, V2, V4, and V6, the length of the heartbeat segment is 5 s, and other steps are similar to the algorithm of arrhythmia detection. During the experiment, each detection device is tested with 128 data. The results are shown in Table 17.

The time ratio is introduced to evaluate the efficiency of the Raspberry Pi in processing the input signal, which is calculated by the ratio of the processing time of the input signal to the length of the input data on the Raspberry Pi. The smaller the time ratio, the faster the device processes the input signal, and if the time ratio is much less than 1, it means that the device can process the signal in real time. For the input signal with a segment length of 9 s, the processing time of the device is 1.16 s, and the time ratio is 0.129. For the detection device of MI, the input signal segment length and the processing time are 5 s and 0.64 s, respectively, and the time ratio is 0.128. Compared with the disease detection process in the PC, the accuracy of the hardware implementation in the Raspberry Pi is 100%, which means that the prediction results on the Raspberry Pi are exactly the same as the prediction results on the PC. This experiment proves that the ECG signal detection device designed in this study can realize the real-time processing of ECG signals with high accuracy. For portable ECG disease detection equipment, it is necessary to consider the resource limitation of the hardware platform, and the algorithm with lower complexity has a tremendous advantage in hardware implementation. This method can flexibly select the algorithm with appropriate complexity under different conditions while ensuring the efficiency of disease detection, which is suitable for hardware implementation and demonstrates its practical application. Moreover, it can select the optimal ECG leads and segment length for different diseases, which reflects its generalization. In conclusion, the experiments verify the convenience of the hardware implementation of the proposed method, which can be used in portable ECG detection devices.

## 4. Discussion

### 4.1. The Analysis of the Results

According to the results, the algorithm provided two different classification frameworks for the diagnosis of cardiac arrhythmias and myocardial infarction, respectively. Premature atrial contractions and premature ventricular contractions in the SH database may be closely related to other cardiovascular diseases such as atrial fibrillation (AF), atrial flutter, myocardial infarction, etc. Therefore, it is meaningful to realize the automatic detection of premature atrial contractions and premature ventricular contractions. Our results (Table 10 and Table 11) show that GA-LSLO provides the optimized lead selection scheme while balancing the classification performance and the algorithm complexity. This also verifies that it is not the case that more ECG leads are better for classification. This may be because, in deep learning, more leads will reduce the sensitivity of the convolutional neural network, and the most important features for disease detection may be submerged in a large number of features, resulting in a decrease in the classification performance of the algorithm. Moreover, the algorithm complexity also increases with the number of leads. Furthermore, when the number of leads is limited (two, three, or four leads), the proposed method can also provide the optimal solution, which is suitable for portable ECG device applications. The ablation experiments in Section 3.5 demonstrate that the LAM can help highlight key leads and improve disease detection performance. Moreover, the weighted cross-entropy loss function improves the detection performance of categories with low quantities. The cross-checking experimental results are consistent with clinical theory, which further verifies the effectiveness of the proposed method. Regarding the utility of the method, the hardware implementation experiments in Section 3.7 demonstrate that the ECG detection device based on the proposed algorithm can achieve real-time processing of ECG signals with high accuracy. The proposed method can provide the most appropriate algorithm with consideration of the resource limitations on the hardware platform while ensuring the efficiency of disease detection, which proves its practical value.

### 4.2. The Comparison with Existing Works

In this section, the classification results of the generated optimal solutions are compared with other existing ECG detection works. Since the detection of arrhythmias is based on the non-public SH database, the comparison of arrhythmia detection is not given here to ensure fairness and the results are only compared with other works in MI detection. Table 18 lists the comparison of MI detection with other works. All results are based on the inter-patient paradigm. 

According to Table 18, the proposed method achieved the highest classification accuracy of 97.62% and F1 score of 96.25% using six leads. Overall, compared with other existing works, the proposed method has obvious advantages. Firstly, it is based on the deep learning model for disease detection and does not require human intervention in feature extraction. Secondly, it automatically optimizes the algorithm through a GA. Selecting the optimal lead and segment length for different types of disease (such as MI and arrhythmia) detection shows strong generalization. Furthermore, the LAM is used in the classification process, highlighting the key lead information to achieve a more accurate disease detection. In addition, the proposed method has lower algorithm complexity than the methods based on all 12 leads [14,46] and is more flexible than the methods with a fixed lead combination [22,24]. In summary, the proposed method can ensure the efficiency of disease detection while optimizing the algorithm with the lowest complexity.

### 4.3. The Contributions

GA-LSLO extracts the features of every single lead under different segment lengths (1–9 s) by a modified ResNet to provide the optimal combination of leads and heartbeat segment length for different disease detection tasks while balancing classification performance and algorithm complexity. The combination of leads and segment length is represented by the encoding strategy proposed in the article, and then the optimal solution is obtained through GA iteration. Compared with other methods using all 12 leads, this method is more flexible and suitable for portable devices.

According to the lead properties of ECG, the lead attention module (LAM) is proposed to capture the dependencies between leads, then update each lead map with lead weights. The LAM is inspired by the channel attention module and it is updated based on the ECG lead properties. Compared with pure multi-layer perceptron (MLP) classifiers, the LAM achieves better performance in heart disease detection.

The generalizability of the algorithm is verified using databases of different disease types. The detection of five categories of arrhythmias in the SH database and the recognition of MI in the Physikalisch-Technische Bundesanstalt (PTB) database obtains good performance. For different databases, the optimal combinations of leads and heartbeat segment length are automatically generated. Moreover, all experiments are based on the inter-patient paradigm, which makes the proposed method more practical and generalizable.

Based on the optimal solutions generated by the genetic algorithm, disease detection devices for arrhythmia and MI are designed using Raspberry Pi, which can realize real-time processing of ECG signals with high accuracy. It is demonstrated that the proposed method has developmental potential and can be implemented in portable ECG devices.

### 4.4. The Limitations

In this paper, the proposed GA-LSLO framework is validated using the SH database and the PTB database, and the results show that it can provide appropriate ECG segment length and lead combination solutions for cardiovascular disease detection tasks. The limitation is that some rare cardiovascular diseases (those that require a long-term monitoring of the patient’s ECG and analysis of the long-term ECG) are not validated. In the future, it will be one of our important works to continue validating the algorithm using more types of cardiovascular disease signals to expand the application of our algorithm. In addition, the hardware implementation is verified on Raspberry Pi, so exploring more hardware platforms is also one of our future works.

## 5. Conclusions

Our proposed GA-LSLO framework can generate the optimal ECG segment length and lead combinations, ensuring the efficiency of disease detection while selecting the algorithm with the lowest complexity. Moreover, ECG detection devices based on the proposed method are realized in Raspberry Pi, which shows the convenience of the hardware implementation and the feasibility of the application on portable devices. In the future, we will apply the algorithm to the detection of other cardiovascular diseases and further explore the field of portable and wearable medical devices.

## Figures and Tables

**Table 1:** Statistics of SH database.

Signal Type	Number of Patients in Training Set	Number of Patients in Test Set
Normal ECG (N)	1336	334
Premature atrial contractions (PAC)	1024	260
Premature ventricular contractions (PVC)	328	82
Tachycardia (T)	532	137
Bradycardia (B)	606	147

**Table 2:** Statistics of the PTB database.

Signal Type	Number of Patients in Training Set	Number of Records in Training Set	Number of Patients in Test Set	Number of Records in Test Set
Healthy controls (HC)	41	63	11	17
Myocardial infarction (MI)	118	294	30	74

**Table 3:** Quantity information of each type of signal at different fragment lengths for the two used databases.

Fragment Length	The SH Database					The PTB Database	
	Number of Fragments					Number of Fragments	
	N	PAC	PVC	T	B	HC	MI
1 s	47,032	33,464	14,553	21,131	17,499	9515	41,455
2 s	22,985	16,458	7169	10,418	8608	4783	20,748
3 s	15,210	10,690	4736	6859	5548	3215	13,945
4 s	11,069	7877	3484	5053	4074	2417	10,388
5 s	8777	6156	2737	3982	3196	1966	8575
6 s	7083	5042	2258	3248	2625	1647	7156
7 s	6030	4139	1889	2732	2129	1407	6087
8 s	5088	3644	1645	2350	1861	1248	5369
9 s	4614	3213	1474	2118	1646	1089	4668

N: normal ECG. PAC: premature atrial contractions. PVC: premature ventricular contractions. T: tachycardia. B: bradycardia. HC: healthy controls. MI: myocardial infarction.

**Table 4:** Detailed configuration information of the feature extraction network.

Layer Name		Number of Filters × Kernel Size	Stride	Activation Function
**Input**		Input size = 1000 (1 s)–9000 (9 s)		
**Conv1+BN**		64 × 13	1	ReLU
**Max Pool1**		—	2	—
**Conv2_x**	Conv2_1+BN	64 × 3	1	ReLU
	Conv2_2+BN	64 × 3	2	ReLU
	Average Pool2	—	2	—
**Conv3_x**	Conv3_1+BN	64 × 3	1	ReLU
	Conv3_2+BN	64 × 3	2	ReLU
	Average Pool3	—	2	—
**Conv4_x**	Conv4_1+BN	128 × 3	1	ReLU
	Conv4_2+BN	128 × 3	2	ReLU
	Average Pool4	—	2	—
**Conv5_x**	Conv5_1+BN	256 × 3	1	ReLU
	Conv5_2+BN	256 × 3	2	ReLU
	Average Pool5	—	2	—
**Conv6_x**	Conv6_1+BN	512 × 3	1	ReLU
	Conv6_2+BN	512 × 3	2	ReLU
	Average Pool6	—	2	—
**Conv7_x**	Conv7_1+BN	512 × 3	1	ReLU
	Conv7_2+BN	512 × 3	2	ReLU
	Average Pool7	—	2	—
**GAP, FC** (Units = 2 or units = 5), **Softmax** (Arrhythmia), or **Sigmoid** (Myocardial infarction)				

**Table 5:** Heart part information reflected by ECG leads.

ECG Leads	Parts of the Heart
I (L_1_), avL (L_5_)	Anterior side wall of the left ventricle
II(L_2_), III(L_3_), avF (L_6_)	Ventricle posterior wall
avR (L_4_)	Inner chamber of ventricle
V1 (L_7_), V2 (L_8_)	Right ventricle
V3 (L_9_), V4 (L_10_)	Ventricular septum
V5 (L_11_), V6 (L_12_)	Left ventricle

**Table 6:** Confusion matrix for SH database.

		Predicted Class				
		N	PAC	T	B	PVC
**True** **Class**	N	998	0	0	0	0
	PAC	1	655	0	0	5
	T	0	0	429	0	0
	B	0	1	0	332	0
	PVC	5	12	0	0	279

**Table 7:** Performance of the SH database.

Class	Sen (%)	Spe (%)	Ppr (%)	Acc (%)	F1 (%)
N	100.00	99.65	99.40	99.78	99.70
PAC	99.09	99.37	98.05	99.30	98.57
T	100.00	100.00	100.00	100.00	100.00
B	99.70	100.00	100.00	99.96	99.85
PVC	94.26	99.79	98.24	99.19	96.21
Average	98.61	99.76	99.14	99.65	98.87

N: normal ECG. PAC: premature atrial contractions. PVC: premature ventricular contractions. T: tachycardia. B: bradycardia. C_1_ = [9,1,1,0,1,1,0,1,1,0,1,0,0].

**Table 8:** Confusion matrix for PTB database.

		Predicted Class	
		HC	MI
**True** **Class**	HC	391	28
	MI	22	1663

**Table 9:** Performance in the PTB database.

Class	Sen (%)	Spe (%)	Ppr (%)	Acc (%)	F1 (%)
HC	93.32	98.69	94.67	97.62	93.99
MI	98.69	93.32	98.34	97.62	98.52
Average	96.01	96.01	96.51	97.62	96.25

HC: healthy controls. MI: myocardial infarction. C_2_ = [5,1,0,1,0,0,1,0,1,0,1,0,1].

**Table 10:** Comparison experiments of 12 single-lead ECG data and all 12 lead ECG data from the SH database.

Lead	Coding	Sen (%)	Spe (%)	Ppr (%)	Acc (%)	F1 (%)
I	[9,1,0,0,0,0,0,0,0,0,0,0,0]	79.58	94.71	77.50	91.93	78.29
II	[9,0,1,0,0,0,0,0,0,0,0,0,0]	81.17	95.17	79.24	92.55	79.73
III	[9,0,0,1,0,0,0,0,0,0,0,0,0]	79.01	94.28	77.66	91.37	78.07
avR	[9,0,0,0,1,0,0,0,0,0,0,0,0]	80.23	95.06	79.55	92.52	79.73
avL	[9,0,0,0,0,1,0,0,0,0,0,0,0]	77.80	93.77	73.83	90.15	74.81
avF	[9,0,0,0,0,0,1,0,0,0,0,0,0]	79.75	94.36	76.37	91.06	77.45
V1	[9,0,0,0,0,0,0,1,0,0,0,0,0]	76.66	93.97	75.84	90.87	76.05
V2	[9,0,0,0,0,0,0,0,1,0,0,0,0]	78.83	94.48	79.40	91.87	78.54
V3	[9,0,0,0,0,0,0,0,0,1,0,0,0]	78.41	94.63	79.38	91.96	78.11
V4	[9,0,0,0,0,0,0,0,0,0,1,0,0]	79.56	94.64	77.45	91.59	77.98
V5	[9,0,0,0,0,0,0,0,0,0,0,1,0]	80.57	94.93	77.63	92.11	78.85
V6	[9,0,0,0,0,0,0,0,0,0,0,0,1]	77.10	94.10	75.19	90.74	75.80
All 12 leads	[9,1,1,1,1,1,1,1,1,1,1,1,1]	97.84	99.68	99.12	99.53	98.41
Proposed	[9,1,1,0,1,1,0,1,1,0,1,0,0]	**98.61**	**99.76**	**99.14**	**99.65**	**98.87**

**Table 11:** Comparison experiments of 12 single-lead ECG data and all 12 lead ECG data from the PTB database.

Lead	Coding	Sen (%)	Spe (%)	Ppr (%)	Acc (%)	F1 (%)
I	[5,1,0,0,0,0,0,0,0,0,0,0,0]	89.41	89.41	93.47	94.68	91.26
II	[5,0,1,0,0,0,0,0,0,0,0,0,0]	82.15	82.15	81.37	88.21	81.75
III	[5,0,0,1,0,0,0,0,0,0,0,0,0]	76.24	76.24	82.78	87.79	78.82
avR	[5,0,0,0,1,0,0,0,0,0,0,0,0]	87.24	87.24	87.72	92.06	87.48
avL	[5,0,0,0,0,1,0,0,0,0,0,0,0]	73.82	73.82	78.25	85.65	75.65
avF	[5,0,0,0,0,0,1,0,0,0,0,0,0]	75.17	75.17	76.75	85.08	75.91
V1	[5,0,0,0,0,0,0,1,0,0,0,0,0]	74.28	74.28	75.54	84.36	74.87
V2	[5,0,0,0,0,0,0,0,1,0,0,0,0]	73.90	73.90	87.18	88.36	78.07
V3	[5,0,0,0,0,0,0,0,0,1,0,0,0]	68.42	68.42	76.02	84.03	70.94
V4	[5,0,0,0,0,0,0,0,0,0,1,0,0]	77.70	77.70	84.08	88.55	80.26
V5	[5,0,0,0,0,0,0,0,0,0,0,1,0]	86.23	86.23	89.78	92.59	87.84
V6	[5,0,0,0,0,0,0,0,0,0,0,0,1]	86.20	86.20	90.09	92.68	87.95
All 12 leads	[5,1,1,1,1,1,1,1,1,1,1,1,1]	93.24	93.24	91.97	95.20	92.59
Proposed	[5,1,0,1,0,0,1,0,1,0,1,0,1]	**96.01**	**96.01**	**96.51**	**97.62**	**96.25**

**Table 12:** The results of disease detection based on a fixed number of ECG leads.

Solutions	SH Database			PTB Database		
	Optimal Lead Combination	Acc (%)	F1 (%)	Optimal Lead Combination	Acc (%)	F1 (%)
Optimal solution	**I, II, avR**, avL, V1, V2, **V4**	99.65	98.87	**I, III, avF**, V2, V4, **V6**	97.62	96.25
Optimal solution fixed with 2 leads	**avR, V4**	90.65	89.48	**I, V6**	95.10	92.29
Optimal solution fixed with 3 leads	**I, avR, V4**	94.63	93.89	**I, avF, V6**	96.10	93.62
Optimal solution fixed with 4 leads	**I, II, avR, V4**	96.95	96.12	**I, III, avF, V6**	96.87	96.13

**Table 13:** Confusion matrix of MI detection using standard cross-entropy loss function.

		Predicted Class	
		HC	MI
**True** **Class**	HC	356	63
	MI	19	1666

**Table 14:** Performance comparison of the weighted cross-entropy loss function.

Loss Function	Sen (%)	Spe (%)	Ppr (%)	Acc (%)	F1 (%)
Cross-entropy	91.92	91.92	95.64	96.10	93.64
Weighted cross-entropy	96.01	96.01	96.51	97.62	96.25

**Table 15:** Results of testing HC and MI data from PTB database using arrhythmia detection model.

		Predicted Class				
		N	PAC	T	B	PVC
**True** **Class**	HC	1081	8	0	0	0
	MI	163	126	2222	0	2157

**Table 16:** Results of testing N, PAC, T, B, PVC data from SH database using MI detection model.

		Predicted Class	
		HC	MI
**True** **Class**	N	8754	23
	PAC	5297	859
	T	27	3955
	B	3174	22
	PVC	146	2591

**Table 17:** Test results for ECG detection devices in this article.

Disease Categories	Segment Length of the Input Signal (s)	Processing Time of Raspberry Pi (s)	Time Ratio	Accuracy of Hardware Implementation
Arrhythmia	9.00	1.16	0.129	100%
MI	5.00	0.64	0.128	100%

Time ratio: processing time of Raspberry Pi/segment length of the input signal. Accuracy of hardware implementation: the number of data for which the Raspberry Pi has the same detection result as PC/total number of test data (128).

**Table 18:** Comparison of existing methods and the proposed method in MI detection.

Research	Database	ECG Leads	Number of Categories	Method	ECG Length (s)	Acc (%)	F1 (%)
[22] 2017	PTB	II	2	CNN	0.651	95.22	-
[24] 2017	PTB	II, III, avF	2	Shallow CNN	3.072	84.54	-
[14] 2019	PTB	All 12 leads	2	SVM	0.8	92.69	83.26
[46] 2020	PTB	All 12 leads	2	MLA-CNN-BiGRU	0.651	96.50	-
Proposed	PTB	I, III, avF, V2, V4, V6	2	GA-LSLO	5	**97.62**	**96.25**

PTB: Physikalisch-Technische Bundesanstalt. CNN: convolutional neural network. SVM: support vector machine. MLA: multi-lead attention. BiGRU: bidirectional gated recurrent unit.

## Data Availability

PTB database is available at https://www.physionet.org/content/ptbdb/1.0.0 (accessed on 20 June 2022). The SH database that has been used is confidential.
